# Supplementary material for: PLZF targets developmental enhancers for activation during osteogenic differentiation of human mesenchymal stem cells
Source: eLife. 2019 Jan 23;8:e40364. doi: 10.7554/eLife.40364 (PMC6344081; doi:10.7554/eLife.40364)
Supplement: Supplementary file 9. [file elife-40364-supp9.docx]

**Supplemental Table 9:** **Oligo Sequences**

| **Primer Name** | **Sequence** | **Used for** |
| --- | --- | --- |
| PLZF_EnP_DpnII | GCTTCTTCTGGTGTGAGATC | 4C-Sequencing |
| PLZF_4C_Csp6I_of_DpnII | CCCTCAACAAAACAATCACT | 4C-Sequencing |
| NNMT_Promoter_DpnII | CACACTTGGTGAGCAGGATC | 4C-Sequencing |
| NNMT_Promoter_Csp6I_of_DpnII | AAGTTGTTATCCTGGTTTGC | 4C-Sequencing |
| Control_Upstream NNMT_DpnII | TTCTTAACCTTACGAAGATC | 4C-Sequencing |
| Control_Upstream NNMT_Csp61 of_DpnII | TACCTCAGCACCTCCTCTTA | 4C-Sequencing |
| Control_Upstream ZBTB16_DpnII | TAGTGAGGGGGCCTAGGATC | 4C-Sequencing |
| Control_Upstream ZBTB16_Csp61of_DpnII | AATGAAGTGGAGACTGAAGG | 4C-Sequencing |
| ZBTB16 Promoter (Primer A) Fwd | ACTAGCGCCCGCGAACT | ChIP |
| ZBTB16 Promoter (Primer A) Rev | CGCCTCCCGGCTTGA | ChIP |
| ZBTB16 Intron 1 (Primer B) Fwd | GTTCTCAGAAACCCCGATGTCT | ChIP |
| ZBTB16 Intron 1 (Primer B) Rev | CAAGGTTGGGATGAGCATTCA | ChIP |
| ZBTB16 Exon2 Fwd (Fig 4A) | AAGGCTCGGTACCTCAAGAACA | ChIP |
| ZBTB16 Exon2 Rev (Fig 4A) | TGTCCAGCCACACTGGCATA |  |
| ZBTB16 Exon3 (Primer C) Fwd | CTGGATAGTTTGCGGCTGAGA | ChIP |
| ZBTB16 Exon3 (Primer C) Rev | GTATCTGAACGGGCCACTAAGG | ChIP |
| ZBTB16 EnP (Primer D) Fwd | TGCCAGCATTCCAGAGAGTTT | ChIP |
| ZBTB16 EnP (Primer D) Rev | CTGCTTTGGGTACAGTGTGTTCTT | ChIP |
| ZBTB16 EnP (Primer E) Fwd | CACCATGGCCTGTTGTAAAATG | ChIP |
| ZBTB16 EnP (Primer E) Fwd | ACAGCAGACCCAAAAAAAGCA | ChIP |
| ZBTB16 Exon6 (Primer F) Fwd | TGGCTGTGGCAAGAAGTTCA | ChIP |
| ZBTB16 Exon 6 (Primer F) Rev | CTGGCCTTCGGTACCTGTGT | ChIP |
| Human PLZF (ZBTB16)_Fwd | GCGGTTCCTGGATAGTTTGC | cDNA-QPCR |
| Human PLZF (ZBTB16)_Rev | TGATCACAGACAAAGGCTTTGG | cDNA-QPCR |
| Human BMP6_Fwd | GATGGCAGGACTGGATCATTG | cDNA-QPCR |
| Human BMP6_Rev | TTGAGTGGGAAGGAGCATTCTC | cDNA-QPCR |
| Human Leptin_Fwd | TGTTGGACACAACAAATTGAAGCAAGC | cDNA-QPCR |
| Human Leptin_Rev | GTGGGTCAATAGAAGGACACATCACG | cDNA-QPCR |
| Human HOPX_Fwd | GAGGAGGAGACCCAGAAATGG | cDNA-QPCR |
| Human HOPX_Rev | TGAGGGCAGGCCTTCTGA | cDNA-QPCR |
| Human OGN_Fwd | GCAAGGCTAATGACACCAGTTACA | cDNA-QPCR |
| Human OGN_Rev | TTCCCAGGACGATTGGATTG | cDNA-QPCR |
| Human CORIN_Fwd | CAGTTGCCCACTGCTTCGA | cDNA-QPCR |
| Human CORIN_Rev | CAAAGCGTGTCTGCATGAACA | cDNA-QPCR |
| Human SPONDIN_Fwd | CGTGAGAGCAGCACCTTCAG | cDNA-QPCR |
| Human SPONDIN_Rev | CTGCAGATAAGCCTACGTTCCA | cDNA-QPCR |
| Human ALPL_Fwd | GGGAACGAGGTCACCTCCAT | cDNA-QPCR |
| Human ALPL_Rev | TGGTCACAATGCCCACAGAT | cDNA-QPCR |
| Human_GAPDH_Fwd | CCACATCGCTCAGACACCAT | cDNA-QPCR |
| Human_GAPDH_Rev | CCAGGCGCCCAATACG | cDNA-QPCR |
| Human NNMT_Fwd | GGCCCTCCCTCTACCATACC | cDNA-QPCR |
| Human NNMT_Rev | AATGATGAAATTCCCGGAGAAG | cDNA-QPCR |
| ZBTB16 SiRNA SASI_Hs01_00148557 | GUUUGAUGACCAUAGGACA[dT][dT] | SiRNAKnocdown |
| ZBTB16 SiRNA SASI_Hs01_00148557-AS | UGUCCUAUGGUCAUCAAAC[dT][dT] | SiRNAKnocdown |
| ZBTB16 SiRNA SASI_Hs01_00148556 | CUGAGAAGCAUCUGGGCAU[dT][dT] | SiRNAKnocdown |
| ZBTB16 SiRNA SASI_Hs01_00148556-AS | AUGCCCAGAUGCUUCUCAG[dT][dT] | SiRNAKnocdown |
| ZBTB16 SiRNA SASI_Hs01_00148555 | CGCAAUAGUCAACACUAUA[dT][dT] | SiRNAKnocdown |
| ZBTB16 SiRNA SASI_Hs01_00148555-AS | UAUAGUGUUGACUAUUGCG[dT][dT] | SiRNAKnocdown |
| GFP-Fwd | TGACCCTGAAGTTCATCTGC | gDNA-QPCR |
| GFP-Rev | GAAGTCGTGCTGCTTCATGT | gDNA-QPCR |
| HOXD locus-Fwd | TTGTTCTCCAGTCGCTTGAC | gDNA-QPCR |
| HOXD locus-Rev | TTCCTCCCTGCCAAACTTTA | gDNA-QPCR |
| EnP-Fwd | CCTGTGTGCTCCCTGTGGGAC | Cloning EnP |
| EnP-Rev | GCTATTCACCAACCTGGCTC | Cloning EnP |
| CtE-Fwd | CTGGCCTTCTTTGTGCTTTCT | Cloning CtE |
| CtE-Rev | TGCTTCTTCTTGCCTTGGTG | Cloning CtE |
| hCOL1A1-ChIP_F | GGGTGGAATCCAGTAGCTGA | ChIP |
| hCOL1A1-ChIP_R | AGGCAATTCCCCTCTTCATT | ChIP |
| hSMAD3-ChIP_F | CCTCTGTGTCCTTCCTCTGC | ChIP |
| hSMAD3-ChIP_R | CGCATACAGCTCAAGGTTCA | ChIP |
| hSMAD3 (CAGE)-ChIP_F | GTGGGAATTTCAGGCACAGT | ChIP |
| hSMAD3 (CAGE)-ChIP_R | AAGAAGGGTGGGGTGCTTAT | ChIP |
| NNMT SiRNA SASI_Hs01_00209920 | SASI_Hs01_00209920 | SiRNAKnocdown |
| NNMT SiRNA SASI_Hs01_00209921 | SASI_Hs01_00209921 | SiRNAKnocdown |
| NNMT SiRNA SASI_Hs01_00209922 | SASI_Hs01_00209922 | SiRNAKnocdown |
